# Real-Time Monitoring of Volatile Compounds Losses in the Oven during Baking and Toasting of Gluten-Free Bread Doughs: A PTR-MS Evidence

**DOI:** 10.3390/foods9101498

**Published:** 2020-10-20

**Authors:** Joana Pico, Iuliia Khomenko, Vittorio Capozzi, Luciano Navarini, Franco Biasioli

**Affiliations:** 1 I.U. Cinquima, Analytical Chemistry Group, University of Valladolid, Paseo de Belén Street 7, 47011 Valladolid, Spain; 2 Research and Innovation Centre, Fondazione Edmund Mach, via E. Mach 1, 38098 San Michele all’Adige (TN), Italy; iuliia.khomenko@fmach.it (I.K.); franco.biasioli@fmach.it (F.B.); 3 Institute of Sciences of Food Production, National Research Council (CNR), c/o CS-DAT, Via Michele Protano, 71121 Foggia, Italy; vittorio.capozzi@ispa.cnr.it; 4 Illycaffè S.p.a, Via Flavia, 110, 34147 Trieste, Italy; Luciano.Navarini@illy.com

**Keywords:** volatile compounds, PTR-ToF-MS, on-line monitoring, gluten-free bread, baking, toasting

## Abstract

Losses of volatile compounds during baking are expected due to their evaporation at the high temperatures of the oven, which can lead to a decrease in the aroma intensity of the final product, which is crucial for gluten-free breads that are known for their weak aroma. Volatiles from fermentation and lipids oxidation are transferred from crumb to crust, and they flow out to the air together with Maillard and caramelisation compounds from the crust. In this study, the release to the oven of volatile compounds from five gluten-free breads (quinoa, teff and rice flours, and corn and wheat starches) and wheat bread during baking and toasting was measured in real-time using proton transfer reaction-time of flight-mass spectrometry (PTR-ToF-MS). Baking showed different volatile release patterns that are described by bell-shaped curves, plateaus and exponential growths. Flour-based breads had the higher overall volatile release during baking, but also high ratios in the final bread, while starch-based breads showed high pyrazine releases due to moisture losses. Meanwhile, toasting promoted the release of volatile compounds from the bread matrix, but also the additional generation of volatiles from Maillard reaction and caramelisation. Interestingly, gluten-free breads presented higher losses of volatiles during baking than wheat bread, which could partially explain their weaker aroma.

## 1. Introduction

Bread is one of the most consumed staple foods all over the world, since it is eaten daily, which makes it an important socio-economic product in human nutrition [1]. Besides its nutritional properties, the sensorial quality of bread undoubtedly determines its consumption, including characteristics, such as volume, texture, colour, and flavour, with the latter understood as the sum of the gustative and olfactory impressions experienced during eating [2]. All of this explains the continuous research activities on how to improve its sensory, chemical, and industrial characteristics [1]. Among these fundamental properties, the aroma of bread plays a key role in its acceptance by consumers [3]. Therefore, the understanding of the generation of volatile compounds during baking as well as their losses due to the high oven temperatures could be essential to ensure an intense and pleasant aroma of the final baked product. The aroma of bread crust has been characterised by volatile compounds from Maillard reactions, caramelisation, and thermal degradation, as well as smaller proportions of volatile compounds from lipid oxidation as a consequence of the cleavage of the hydroperoxides at high temperatures [4]. However, there can also be migrations of volatile compounds from the crumb to the crust during baking, which leads to the presence in the crust of compounds from fermentation, Ehrlich pathway, and non-enzymatic lipids oxidation that are common in the crumb.

Focusing on gluten-free breads, the sensory quality, especially its crust aroma, has been defined as barely acceptable [5], even though gluten-free breads are essential in the diet of coeliac people. Some attempts to improve the aroma of gluten-free bread crusts have been made so far, including the addition of amino acid-sugar pairs in order to promote Maillard reaction [6] or the volatile profile evaluation of baked crusts of bread from different gluten-free ingredients for more pleasant aromas [4]. However, potential losses of volatiles during gluten-free bread baking have never been explored as a possible reason for the less intense aroma as compared to the conventional wheat bread. Consequently, the on-line monitoring of the volatile compounds produced and released in real-time during baking could help to understand and improve the weaker aroma of gluten-free breads. There have been some attempts to understand the release of volatile compounds to the oven during the baking of wheat bread [7]; however, they employed Solid-Phase Microextraction (SPME) cartridges that adsorb/absorb volatiles that escaped from the oven for a further desorption in the GC/MS injector, eliminating the real-time component of the process and biasing which compounds are adsorbed/absorbed based on the nature of the cartridge. Consequently, analytical techniques that allow for the real on-line monitoring of the vapours naturally escaping from the bread to the oven are needed, which can be achieved using Proton transfer reaction-time of flight-mass spectrometry (PTR-ToF-MS).

PTR-ToF-MS is a direct-injection mass spectrometric technique that is based on the proton transfer reaction from hydronium ions (H_3_O^+^) to those compounds with larger proton affinities than water, mainly producing protonated molecular ions MH^+^, but also smaller fragments, e.g., from the dehydration-protonation of tertiary alcohols, aldehydes, acids, and esters [8]. PTR-ToF-MS has already been successfully applied to the characterisation of volatile compounds in baked wheat bread for the evaluation of its freshness [9], the effect of bakery yeasts and flours in wheat doughs and baked breads [4,10,11], the retronasal aromas released from the nose-air of consumers during the oral processing of wheat breads [12,13,14], and even for the aroma of baked and toasted crumbs and crusts of gluten-free breads as final products [15]. However, PTR-ToF-MS has never been applied to the on-line monitoring of the vapours released into the oven from the bread dough during baking, which certainly would contribute to the understanding of the aroma intensity of the final product. The evaluation of the on-line monitoring of gluten-free bread doughs aroma during baking and toasting could be essential to understand whether losses of volatile compounds in the oven are the cause of their weak aroma and it has never been investigated in detail previously.

Therefore, the main aim of this study was to on-line monitor, for the first time, the release of volatile compounds from five gluten-free doughs (quinoa, teff and rice flours, and corn and wheat starches) and wheat bread dough during their baking and toasting processes. For this purpose, the volatile compounds that were emitted from the dough to the oven during baking and toasting were analysed by the PTR-ToF-MS instrument that was directly connected to the oven.

## 2. Materials and Methods

### 2.1. Materials and Standards

2-acetyl-1-pyrroline was purchased from Eptes (Vevey, Switzerland), while the rest of pure standards of Table 1 (labelled from 1 to 39) were obtained from Sigma–Aldrich (Steinheim, Germany), all with a purity > 98%. Dichloromethane was obtained from Scharlab (Barcelona, Spain) and methanol from VWR International (Fontenay-sous-Bois, France). Argon was acquired from Carburos Metálicos (Barcelona, Spain).

### 2.2. Preparation of Standard Solutions

2-acetyl-1-pyrroline solutions were prepared in dichloromethane, working under an inert atmosphere of argon at all times due to the lack of stability of the compound to oxygen and moisture. Consequently, dichloromethane was dried in an SDS PS-MD-5 purification system from Düperthal Sicherheitstechnik (Karlstein am Main, Germany). The other 38 volatile compounds, which are included in Table 1, were prepared in methanol. All of the solutions were stored in a freezer at −21 °C.

### 2.3. Gluten-Free and Wheat Bread Ingredients

Wheat starch was purchased from Paneangeli (Desenzano del Garda, Italy) and corn starch from Unilever (Roma, Italy). Wheat flour was acquired from Coop (Casalecchio di Reno, Italy), quinoa flour and wholemeal teff flour from Ecor (Schio, Italy), and wholemeal rice from Ki (Torino, Italy). Hydroxyl propyl methyl cellulose (HPMC) was supplied by Dow Chemicals (Milan, Italy) and the dry baker’s yeast (*Saccharomyces cerevisiae*) was obtained from Lesaffre (Cerences, France).

### 2.4. Gluten-Free Bread Making

The following ingredients were mixed while using a Princess machine model 152,006 (Taunton, MA, USA) for 1 min: starch or flour (200 g), sunflower oil (12 g), sucrose (10 g), salt (3.6 g), HPMC (4 g) and water (75 g). The yeast (3 g) was rehydrated in 25 g of water, added to the rest of the ingredients, and then mixed for another 15 min. The fermentation of the dough (250 g) took place in a chamber Climacell 707 model CLC-B2V ( Planegg, Germany) for 90 min, at 30 °C and 90% of relative humidity, using PTFE-coated containers of 250 × 110 × 35 mm covered by baking paper. Afterwards, the dough was placed in an oven Binder model WTB (Tuttlingen, Germany) that was directly connected to the PTR-ToF-MS through a PEEK capillary tube inlet (internal diameter 1 mm) heated at 110 °C. Thus, the baking vapours (headspace) were on-line monitored at 200 °C for 40 min. baking. The two central slices of 3 cm width were cut from each bread right after baking and placed again in the oven for the on-line monitoring of the toasting vapours for 20 extra minutes at 200 °C in order to simulate the toasting step. Each dough was prepared in triplicate and its baking/toasting was monitored on-line.

### 2.5. On-Line Monitoring of Volatile Compounds during Baking and Toasting of Gluten-Free and Wheat Control Breads by PTR-ToF-MS

The on-line monitoring of the volatile compounds from the headspace generated during the baking and toasting of the breads made with different gluten-free sources and wheat flour bread (control) was performed with a PTR-ToF-MS 8000 apparatus from Ionicon Analytik GmbH (Innsbruck, Austria), in its standard configuration (V mode), which was directly connected to the baking oven. The ionisation conditions in the drift tube implied 110 °C of drift tube temperature, 2.30 mbar of drift pressure, and 550 V of drift voltage, which led to an E/N ratio of about 140 Townsend. The PTR-ToF-MS inlet flow controller (FC) was set up at 340 standard cubic centimetres per minute (sccm) and the acquisition rate was 1 spectrum/s. Because of the presence of relevant amounts of ethanol in the doughs as a consequence of the alcoholic fermentation, their headspace was 1.8-fold diluted with the constant flow of 270 sccm of zero air generated by a Gas Calibration Unit apparatus (Ionicon Analytik GmbH, Innsbruck, Austria), which was connected to the inlet system immediately outside from the oven.

Once the mass peaks (*m*/*z*) from impurities, clusters, and isotopes were excluded, the tentative identification of possible molecular formulas was performed while using a mass calculator. For this purpose, those formulas that gave a lower error in the mass calculator were inserted in the NIST library (as neutral formulas, considering both the protonated and the dehydrated-protonated ions), giving different compounds that fit with the formula. Based on the literature about bread aroma, one or more different compounds were suggested as possible volatile compounds corresponding for each *m*/*z* (Table 1). The *m*/*z* corresponding to 39 volatile compounds selected as important contributors to bread aroma were confirmed through the individual injection of pure standard in the fast-GC-PTR-ToF-MS system (Supplementary Table S1). The thirty-nine standards were injected using a multipurpose autosampler model MPS from Gerstel (Mulheim am Main, Germany), which was adapted to the PTR-ToF-MS [16]. Before the measurements, the standards were kept in trays at 4 °C. The headspace analysis took place at 40 °C for 30 s in order to fill the fast GC sampling loop. The polar capillary column [MXT^®^-WAX (Siltek^®^—treated stainless steel), 6 m] was maintained under pure N_2_ with a constant flow rate of 3 sccm. The chromatographic total run time was 150 s, with a thermal ramp from 40 °C to 220 °C at 2.25 °C/s and holding at 220 °C for 70 s. The conditions of the drift tube were as follows: drift voltage 630 V with the ion funnel, drift temperature 110 °C, and drift pressure 2.80 mbar, resulting in an E/N value of about 130 Townsend. The data acquisition rate was set to 5 spectra/s. It should be noted that the on-line monitoring of the bread baking vapours implied the direct connection of the oven to the PTR-ToF-MS, thus there was not chromatographic separation. The purpose of the standards injection in the fast-GC-PTR-ToF-MS system was the identification of the MS profile (i.e., *m*/*z*) of the selected compounds.

### 2.6. Bread Moisture Loss

Bread moisture loss (%) was evaluated 1 h after baking. It was calculated as the weight difference of the bread before and after the baking process.

### 2.7. Statistical Analysis

The raw PTR-ToF-MS data were processed and analysed while using self-developed routines in MATLAB (MathWorks, Natick, MA, USA). The retention time of fast-GC PTR-ToF-MS peaks was calculated using the core R libraries and external libraries Peaks and MESS (R Foundation for Statistical Computing, Vienna, Austria).

## 3. Results and Discussion

### 3.1. On-Line Monitoring of Volatile Compounds during Bread Baking by PTR-ToF-MS: General Patterns

**Figure 1:**
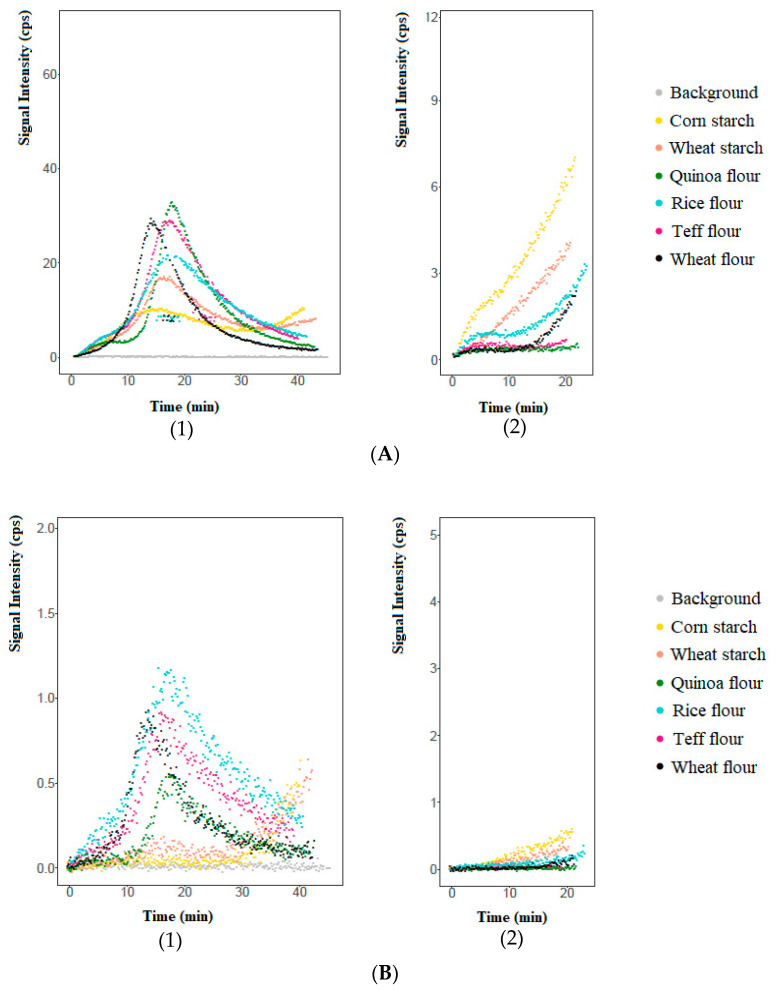

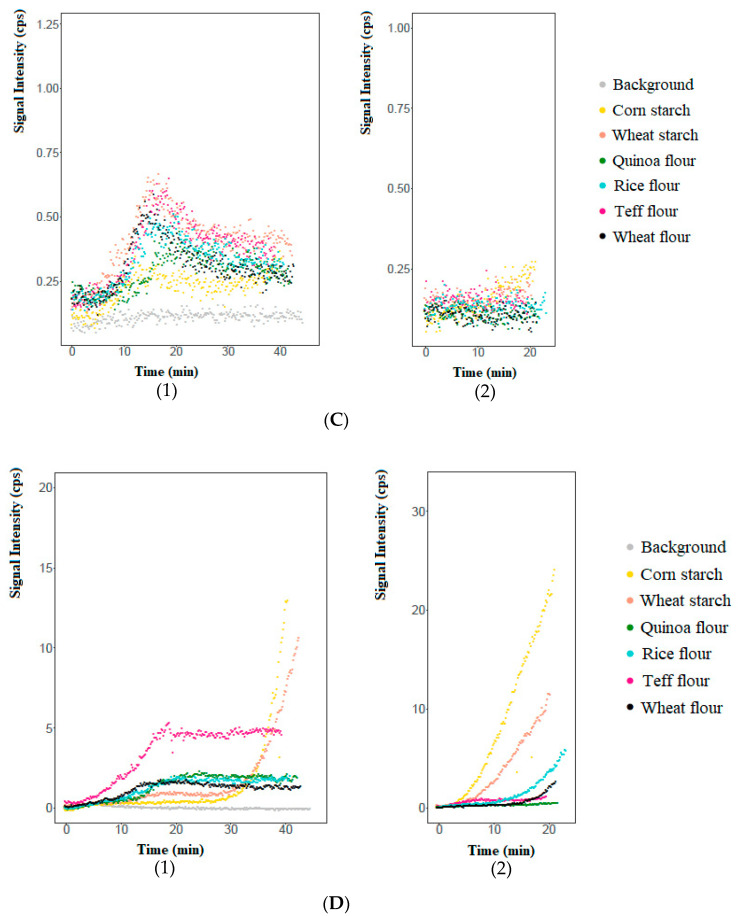

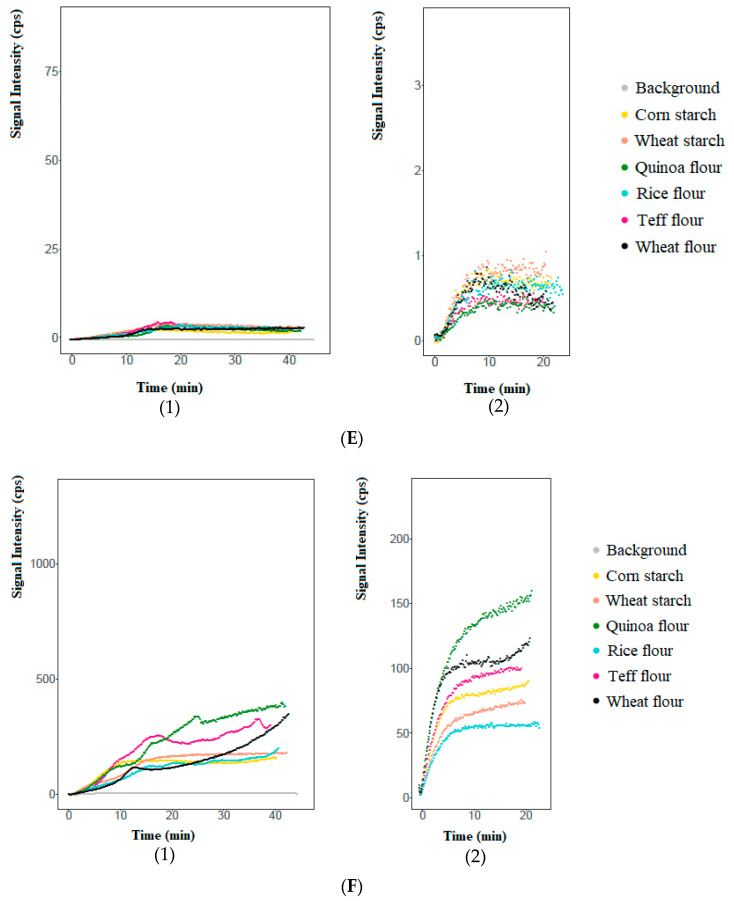

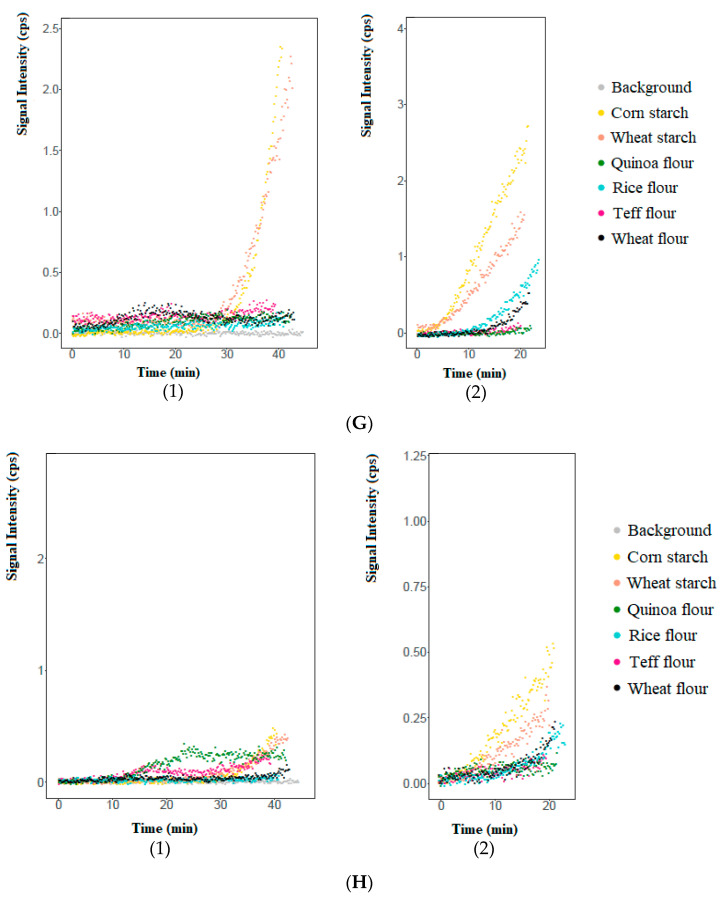

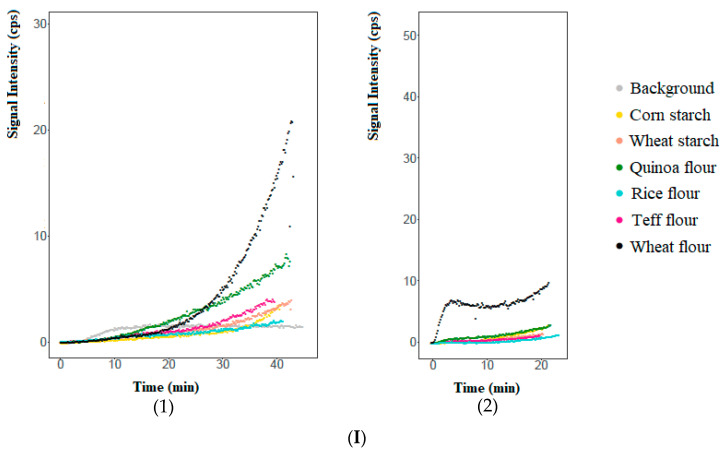
PTR-ToF-MS on-line curves of the volatile compounds released during 40 min. of baking (**1**) and further 20 min. of toasting (**2**). *X*-axis shows the time of baking/toasting, while *Y*-axis shows the signal intensity (cpu). Data corresponding to one compound were reported as an example of each of the 9 groups (classified by their biological origin): 3-methyl-1-butanol (fermentation origin, group 1, **A**), 1-hexanol (lipids oxidation origin, group 2, **B**), ethyl octanoate (esters origin, group 3, **C**), hexanoic acid (group 4, **D**), phenylethyl alcohol (fermentation-Maillard high retention in bread origin, group 5, **E**), acetic acid (fermentation-Maillard low retention in bread origin, group 6, **F**), 2,3-diethylpyrazine (early Maillard origin, group 7, **G**), 2-acetyl-1-pyrroline (late Maillard origin, group 8, **H**), furfuryl alcohol (caramelization-Maillard origin, group 9, **I**).

259 peaks were on-line monitored by means of PTR-ToF-MS in the vapours release to the oven from the dough of six different breads (corn and wheat starch and quinoa, teff, rice and wheat flours) during 40 min. baking. After deleting peaks from the background and removing isotopes, 86 peaks were tentatively identified based on their molecular formula from the NIST database and the literature of food aroma. Finally, 54 volatile compounds were selected as important contributors of bread aroma [17], including alcohols, aldehydes, ketones, acids, esters, terpenes, furans, pyrroles, pyrazines, and the presence of 39 of them was verified examining the presence of the *m*/*z* found with standards that were injected by fast-GC-PTR-ToF-MS (Supplementary Table S1). The total amount of each volatile compound released by each dough during the 40 min. of baking, based on the corresponding *m*/*z* (Table 1), was depicted in Figure 1A–I. Among the PTR-ToF-MS curves of the release of volatile compounds, nine patterns were observed according to volatiles formation origin (regardless of the type of flour or starch). These patterns are summarised in Table 2, classified by nine groups, including: (1) fermentation origin, excluding esters, which are volatiles exclusively generated by fermentation; (2) lipids oxidation origin, which are volatiles generated exclusively by enzymatic and non-enzymatic lipids oxidation; (3) esters origin, generated through reactions catalysed by acetyltransferases in the yeast cell or through lipids oxidation by the action of acetyl transferases over the alcohols; (4) acids origin, generated by fermentation and lipids oxidation; (5) fermentation–Maillard high retention in bread origin, comprising compounds generated through both sources, including some acids; (6) fermentation–Maillard low retention in bread origin, comprising compounds generated through both sources, including some acids; (7) early Maillard origin, including compounds exclusively generated by Maillard that needed less time in the oven to be formed; (8) late Maillard origin, including compounds exclusively generated by Maillard that needed more time in the oven to be formed; and, (9) Caramelization–Maillard origin, composed volatiles generated not only by Maillard, but also by caramelisation reactions.

Volatile compounds that belong to the group 1 (fermentation origin, Figure 1A, 3-methyl-1-butanol) have been reported to be mainly generated by fermentation [17,18] and it tentatively includes 3-methyl-1-butanol, 2-methyl-1-butanol, 1-pentanol, 2,3-butanedione, acetaldehyde, and ethanol (see *m*/*z* in Table 1). Their release curve followed a bell-shaped curve. These compounds were continuously released from the dough to the headspace of the oven between 0 and 10–20 min, reaching the maximum and decreasing afterwards to values near to zero at 40 min. It can be concluded that these compounds were released and they accumulated in the first 10–20 min. with the following decrease up to initial values due to the extraction by the PTR-ToF-MS or the oven exhaust. Only for breads that were prepared with starches, there was again a small release of these volatiles in the last couple of minutes of baking. This can be attributed to the higher moisture loss (34.95% for quinoa bread, 35.88% for teff bread, 25.34% for rice bread, 27.00% for wheat bread, 38.22% for corn starch bread, and 38.84% for wheat starch bread) and drier crumbs of cereal starch breads versus cereal flour breads, which leads to the leakage of volatile compounds flowing out together with the water during baking [5]. Therefore, it is very likely that compounds from group 1 were produced in the dough by the action of the yeast during the fermentation step and they later transferred from the crumb to the crust and, then, to the headspace of the oven during baking. This origin is in accordance with the evaporation from dough to crumb reported for 3-methyl-1-butanol, 2-methyl-1-butanol, and 2,3-butanedione in corn starch bread [19], as they have been demonstrated to be highly released to the oven during our PTR-ToF-MS measurements of baking. In spite of being released in great amounts to the oven, volatile compounds from fermentation have been reported to be the main compounds in breads crumbs analysed by PTR-ToF-MS [15], since the temperatures that are reached in the crumb during baking are not enough for a relevant generation of other types of volatile compounds.

Volatile compounds belonging to group 2 (lipids oxidation origin, Figure 1B, 1-hexanol) have been reported to be mainly generated by lipids oxidation [17,18] and it tentatively includes 1-hexanol, hexanal, heptanal, 1-octen-3-ol, nonanal, 2,4-decadienal (see *m*/*z* in Table 1). Lipid oxidation volatile compounds can be produced by non-enzymatic reactions during kneading and fermentation or by the generation of hydroperoxides through the action of lipoxygenases during kneading and fermentation that are broken later at high temperatures during baking [20]. Thus, volatiles from breads that are prepared with cereal flours presented similar patterns to the group 1 (following a bell-shaped curve), although in a much lower proportion, being released and accumulated in the first 10–20 min and simultaneously extracted from the headspace by the PTR-ToF-MS or through the oven exhaust until the end of baking. On the one hand, this leads to the conclusion that they were mainly generated and accumulated in crumb prior to baking by non-enzymatic reactions during kneading/fermentation, since they appeared in the headspace of the oven at the very beginning of baking (while hydroperoxides from enzymatic reactions would need more time at the high oven temperatures to be broken in the crust). On the other hand, it is also possible to conclude that their generation in the crust through the break of the hydroperoxides should be limited, or at least lower, than the speed of extraction by PTR-ToF-MS/oven exhaust; otherwise, the curve should always be increasing instead of reaching a maximum and later decrease. Taking this into consideration, the very late release of lipid oxidation volatile compounds in breads that were prepared with starches could be due to their more active generation by lipoxygenases, which might need more time to be accumulated in the crust and released to the oven (also considering that starches present very low lipids content when compared to cereal flours). If non-enzymatic reactions from kneading/fermentation would have taken place in starches dough, then the corresponding lipids oxidation volatile compounds would have been released at the beginning of baking due to evaporation. All of this is in accordance with Pico, Martinez et al. (2017) [19], who reported that these volatile compounds are present in the dough, but also increase their concentration during baking with an accumulation in the crumb.

To the group 3, mainly belongs esters, which are developed during fermentation [18]. Esters have been reported as commonly lost during baking and it has usually been attributed to their hydrolysis catalysed by the heat during baking [21]. Ethyl octanoate, ethyl hexanoate and hexyl acetate (esters origin, Figure 1C, ethyl octanoate) were tentatively detected by PTR-ToF-MS in the headspace of the oven in very low ratios (see *m*/*z* in Table 1). They presented a similar pattern of a bell-shaped curve than other volatile compounds ascribable to fermentative process (group 1).

The mass 117.0921, tentatively identified as hexanoic acid, has an exclusive origin belonging to group 4 (Figure 1D) that can be caused by the evidence that was reported in the literature concerning a possible generation both by fermentation and lipids oxidation [17]. During the first 10–20 min, hexanoic acid from breads that were prepared with cereal flours presented a similar origin to the group 2 and it was released up to close levels, which can be attributed to its generation by fermentation or non-enzymatic lipid oxidation reactions and the subsequent release in the oven at high temperatures. However, instead of reaching a maximum and later decrease, hexanoic acid kept a plateau. This suggests that there is an equilibrium between the generation of hexanoic acid (through the breaking of the hydroperoxides) and the extraction of molecules from the headspace by PTR-ToF-MS and/or their exhaust out the oven. Furthermore, as it happened for group 2 with breads that were prepared with starches, there was a very late release that seems to be in accordance with its generation through the breaking of hydroperoxides during baking.

Groups 5 and 6 comprise volatile compounds that are the result of both fermentation and Maillard reactions, with the last taking place with the high temperatures of baking. Group 5 (fermentation–Maillard high retention in bread origin, Figure 1E, phenylethyl alcohol) is tentatively constituted by phenylethyl alcohol, phenylacetaldehyde, benzaldehyde, 3-methyl-butanoic acid, and 2-methyl-butanoic acid (see *m*/*z* in Table 1), while group 6 (fermentation–Maillard low retention in bread origin, Figure 1F, acetic acid) is tentatively composed by acetic acid, butanoic acid, and acetoin (see *m*/*z* in Table 1). The distinction between both groups is based on the ratio of volatile compounds that escaped to the oven, which is a balance between the amount that is produced in the bread and their interaction with matrix compounds from crumb and crust that hindered their release to the headspace. On this basis, volatile compounds from group 5 could have been released to the oven in lower amounts, due to higher retentions in the bread matrix, while volatile compounds from group 6 released in higher amounts could have been poorly retained bread. The volatiles retention in bread is undoubtedly related to the physicochemical interactions with the bread matrix and it is a key factor on the flavor perception [22,23]. On the one hand, fat has been explained to significantly interact with hydrophobic volatile compounds [23], while starch is known to interact with polar volatiles through complexes with amylose [22,23]. On the other hand, protein interactions with volatile compounds have been considered to be weaker than the interactions of volatiles with fat [23]. Therefore, the higher retention of volatiles from group 5 could be partially explained by their stronger interaction with fat based on their higher hydrophobicity when compared to acetic acid, butanoic acid, and acetoin from group 6. Finally, the curve again implied an increase up to 10–20 min. and a further plateau (exponential plateau), except for acetic acid in quinoa, teff and wheat bread that slightly increased. This is in line with the generation of volatiles during fermentation and subsequent release in the oven during the first minutes of baking, followed by a permanent generation and release of Maillard compounds until the end of baking (which is in equilibrium with the extraction by PTR-ToF-MS or the oven exhaust).

Volatile compounds that were exclusively generated by Maillard and related reactions (i.e., Strecker aldehydes) belong to groups 7 and 8. The splitting between these groups is based on the stage of baking that their volatile compounds, mainly in the case of starch breads, are detected in the headspace of the oven. The higher release to the oven in starch breads could be attributed to their higher moisture loss as compared to cereal breads (as it was explained for group 1), as well as to their possible higher generation during baking considering their reported higher amount in the final crust of wheat starch breads that were prepared with a similar formula, in spite of their lighter colour compared to cereal breads [15]. Thus, volatiles are from group 7 (early Maillard origin, Figure 1G, 2,3-diethylpyrazine) if they started to be visibly released in starch breads after 30 min. (tentatively were 1-methyl-pyrrol, limonene, 3-methylbutanal, 2-methylbutanal, 2,3-diethyl-5-methylpyrazine, 2-ethyl-3-methylpyrazine, 2-acetylpyrazine, 2,3-diethylpyrazine, 2,5-diethylpyrazine, 2,6-diethylpyrazine, 3-ethyl-2,5-dimethylpyrazine, 2-ethyl-3,5-dimethylpyrazine, 5-ethyl-2,3-dimethylpyrazine, 3-ethyl-2,6-dimethylpyrazine, and 2-ethyl-3,6-dimethylpyrazine, see *m*/*z* in Table 1), while they are from group 8 (late Maillard origin, Figure 1H, 2-acetyl-1-pyrroline) if they were scarcely released from starch breads after 35 min. (tentatively were 2-acetyl-1-pyrroline, pyrazine, 2-ethylpyrazine, 2,3-dimethylpyrazine, 2,5-dimethylpyrazine and 2,6-dimethylpyrazine, see *m*/*z* in Table 1). Maillard reaction needs temperatures of 110–150 °C to take place, while the bread surface needs at least 20 min. to reach 180–190 °C if the oven is at 220 °C [24]. Therefore, it is coherent that at least 20 min. are necessary for observing a release of Maillard compounds to the oven and their subsequent detection by PTR-ToF-MS. Their release profile also followed the shape of an exponential growth. However, the levels of volatiles detected is very little for all kind of breads, especially for cereal breads from both groups and starch breads from group 8, regardless the time of baking at which PTR-ToF-MS detects these Maillard compounds. Higher levels of pyrazines would have been expected to be released from breads when considering that concentrations between 9.5 and 141 μg Kg^−1^ were reported in corn starch bread crust prepared with a similar recipe [4] and PTR-ToF-MS has been characterised by limits of detection of parts per trillion (ppt) [25]. Therefore, it seems that these Maillard compounds were not easily released after their generation, or they needed more time to be greatly generated and reach the headspace (as it will be demonstrated during toasting).

Finally, group 9 (caramelization–Maillard, Figure 1I, furan) is formed by volatile compounds that are generated by both caramelisation and Maillard reaction and it tentatively includes furan, furfuryl alcohol, furfural, 5-methyl-2(5H)-furanone, 5-methyl-furfural, 5-Methyl-2(5H)-furanone, 2-acetylfuran, and 3-penten-2-ol (see *m*/*z* in Table 1). Caramelisation happens at higher temperatures than Maillard, between 150 °C and 200 °C [24], thus both processes take place in the crust after around 20 min at the temperatures of the oven. Thereby, the detection of these compounds started around 20 min and they were released in much higher ratios than Maillard compounds from groups 7 and 8, also following the curve shape of an exponential growth. This is in accordance with previous analyses of furans in bread crusts by PTR-ToF-MS [15], where furans were in much higher concentration than pyrazines since furans have a double source of generation. Therefore, despite being released in a bigger amount to the headspace, a great part of furans also remains in the crumb.

### 3.2. On-Line Monitoring of Volatile Compounds during Bread Toasting by PTR-ToF-MS: General Patterns

The toasting of bread was made using two central slices of baked bread of 3 cm each one, which were placed again in the oven for 20 extra minutes. The toasting step was performed in a way that mimics the commercial and domestic toasting process, which never implies the toasting of the whole bread. Group 4 (hexanoic acid, Figure 1D) presented higher releases that even the whole baked bread, while the group 7 (early Maillard, Figure 1G), 8 (late Maillard, Figure 1H), and 9 (caramelization-Maillard) presented similar releases than the latter, considering that during toasting the amount of bread of two slices was always smaller. On this basis, it could be concluded that the breaking of the hydroperoxides that gave rise to hexanoic acid as well as the Maillard (groups 7 and 8) and caramelisation reactions (group 9), needed more than 40 min. at 200 °C to be generated and/or released in greater amounts. Therefore, the extent of the release during toasting depends on the pathway of generation, namely Maillard reaction and caramelisation, which need high temperatures, and on the level of interaction of the already generated compounds with the matrix. The rest of volatile compounds from fermentation and non-enzymatic lipids oxidation presented smaller releases than during baking, which can also be attributed to the smaller amount of bread present in the oven. However, regardless their extent, all the volatile compounds presented a continuously release during toasting, which can be promoted by the disruption of the matrix during baking (i.e., starch gelatinisation, proteins coagulation, gas cell formation) that weaken the interaction of the volatiles with the matrix, promoting their release. Thus, the continuous extraction by PTR-ToF-MS was only possible if there was a continuous release from the matrix.

In conclusion, toasting promoted the substantial generation and/or release of volatile compounds whose main source was Maillard and caramelisation reactions, while those compounds that were mainly produced before baking (fermentation and non-enzymatic lipids oxidation) experimented a small additional release as a consequence of the modified matrix.

### 3.3. Differences in the Extent of Release of Volatile Compounds during Baking and Toasting Based on the Type of Flour: Gluten-Free and Wheat Control Breads

Five different breads from diverse gluten-free ingredients (quinoa flour, teff flour, rice flour, corn starch, and wheat starch) and wheat flour bread (control sample) were on-line monitored for the analysis by PTR-ToF-MS of the volatile compounds released to the oven during 40 min. of baking and 20 min. of toasting at 200 °C. The level of emission of volatile compounds to the oven would depend on their amounts already generated in the bread during fermentation (including Ehrlich pathway), lipids oxidation, Maillard reaction (including Strecker degradation), and caramelisation, as well as the interaction of these volatile compounds with the bread matrix that would hinder their release to the headspace. Because the recipe is the same for the six breads, the difference in the generation and/or release of volatile compounds to the headspace should be attributed to the variable ingredients employed (quinoa, teff, rice and wheat flours, as well as corn and wheat starches), as sources of both precursors of volatiles and molecules that interact with volatile compounds.

Regarding the group of baking “fermentation origin” (group 1), with 3-methyl-1-butanol by way of example (Figure 1A), “maximum value of the peak” and “time to reach the maximum” should both be considered. The maximum achieved by the peaks followed the order quinoa flour > teff flour ~ wheat flour > rice flour > wheat starch > corn starch. Higher levels of volatiles in the oven headspace could be partially attributed to higher amounts of these compounds generated in the bread. Thus, the higher levels of volatiles from fermentation that were generated by the yeast metabolism in quinoa bread could be ascribed to its reported higher amylolytic enzymatic activity regarding other flours [26], which brought out to more glucose available for the yeast. Volatile compounds from fermentation like 3-methy-1-butanol have been positively related to the final aroma bread [17], leading to the conclusion that both the aroma emitted by quinoa bread to the air as well as the aroma retained in quinoa bread crumb were pleasant in terms of these volatile compounds. Wheat flour-, corn starch-, and wheat starch-based breads reached the maximum release of fermentation compounds (group 1) that were remarkable before than the rest of breads, which could be related to the higher moisture loss that helped the volatiles to leak to the headspace in the case of starches (as explained before). However, wheat flour bread presented one of the lowest moisture loss values, thus the earlier release of volatiles could be attributed to other factors, such as the lower volatile retention capability of wheat flour crust versus rice or teff flour crusts when they tried to escape to the headspace. Indeed, the concentration of 3/2-methyl-1-butanol and 2,3-butanedione in wheat flour bread crust (following our same recipe) was reported as 17.99 μg Kg^−1^ and 4.98 μg Kg^−1^, while, for teff flour bread crust, it was 19.34 μg Kg^−1^ and 19.10 μg Kg^−1^, respectively [4]. Thus, it is hypothesised that ‘teff’ crust was able to better retain these compounds when they were transferred from the crumb to the crust and later to the air.

Regarding the group of baking “lipids oxidation origin” (group 2), with 1-hexanol as an example (Figure 1B), they also present “maximum value of the peak” and “time to reach the maximum” as fermentation compounds. The maximum achieved by the peaks follow the order rice flour > teff flour ~ wheat flour > quinoa flour > wheat starch > corn starch. Quinoa has been reported to present higher levels in lipids than the other used flours [27], but also higher amounts of antioxidants (i.e., vitamin E and flavonoids) [28,29] and lower lipoxygenase activities [30] that inhibit the generation of lipids oxidation volatile compounds. This explained the reason why ‘quinoa’ bread showed the lower content of these volatile compounds, as has been previously reported for quinoa bread crumb [31]. Lipid oxidation volatile compounds, such as hexanal or 2,4-(E,E)-decadienal, have been negatively correlated with the perceived aroma of bread, which means that the aroma that was released by ‘teff’ and ‘rice’ bread was negatively influenced by these volatile compounds. Meanwhile, the low amount of lipids in pure starches explained their low content of lipids oxidation volatile compounds as well. On the other hand, ‘rice’ and ‘teff’ crust have been already reported to have a similar profile than wheat flour crust in terms of higher contents of lipids oxidation volatile compounds [4], justifying the order in the contents of these compounds. In regard to the “time to reach the maximum”, the earlier release of wheat bread follows the same pattern than fermentation volatile compounds from group 1, which supports the theory of the lowest retention in crust by wheat flour bread.

Esters from group 3 showed such a small release to the headspace that there were no clear differences in content between the different breads. Regarding hexanoic acid from group 4, it was released to the oven in significantly higher amounts by teff bread from 0 to 20 min., reaching the plateau after 20 min. Meanwhile, ‘starch’ breads needed 30 min. to start the exponential release of hexanoic acid and they did it, even at higher final levels than those of teff bread (following the same pattern than bread starches from group 2). Assuming that hexanoic acid interacts similarly with the matrix in all types of breads, its higher level in the headspace of ‘teff’ bread can be attributed to the common higher level in lipids oxidation volatile compounds reported for this bread [32,33].

Volatile compounds from the baking group 5 (fermentation–Maillard highly retained in bread) were released in such a small ratio that they were similar in all kind of breads, while for group 6 (fermentation–Maillard low retained in bread) volatiles were only released in higher levels for quinoa bread. This could be partially attributed to the larger generation of acetic acid and acetoin in ‘quinoa’ bread regarding ‘teff’,’ rice’ or ‘wheat’ flour breads and ‘corn starch’ bread, which has been already verified in the aroma of the final crumb from breads prepared with the same recipe than the present study [33]. In line with fermentative volatile compounds from group 1, which were also presented in bigger amounts in the headspace of ‘quinoa’ bread, volatile compounds from groups 5 and 6, such as phenylethyl alcohol or acetoin, have also been characterised by pleasant aromas [17].

Maillard compounds from groups 7 and 8 were detected after 40 min of baking in considerably higher levels in the headspace of starch-based breads, regardless of whether the release started at 30 min (group 7) or 35 min (group 8). The total content (as the sum of crumb and crust) of pyrazine and dimethyl-pyrazines, as measured in previous studies of the research group by PTR-ToF-MS, was reported to be in higher concentrations for quinoa and wheat flour breads, while ‘starch’ breads exhibited values close to the average, specially wheat starch bread [15]. Only the content of dimethyl-pyrazines and ethyl-dimethyl-pyrazines measured by PTR-ToF-MS was usually smaller in ‘starch’ breads [15]. Therefore, the highest concentration of pyrazine derivatives has not been reported to be in starch breads, although the amounts were still significant. When considering this, the higher release of these volatile compounds in starches could be attributed to the higher moisture losses in these breads, as was explained before. Pyrazine derivatives have been described as one of the main responsible of the pleasant aroma of bread crust [4], which can explain the nice aroma release from the oven during the baking of ‘starch’ breads (although this could lead to the depletion of these volatiles in starch breads). Finally, furan derivatives from group 9 were released in much higher ratios in wheat and quinoa flour breads, which is in full agreement with their higher total content measured by PTR-ToF-MS (sum of crumb and crust) in our previous studies [15].

The high levels of caramelisation compounds in the headspace of ‘quinoa’ bread, as well as the high levels of pleasant volatile compounds from fermentation (groups 1, 5, and 6) and the lower ratios of off-flavours from lipids oxidation (group 2), makes of quinoa flour a very suitable candidate for the improvement of the perceived aroma of gluten-free bread (i.e., aroma released to the air) when heated at high temperatures. This is in accordance with the PTR-ToF-MS results of the aroma of baked quinoa bread crumb and crust (static experiment of the final product) that were previously reported by the research group [15]. Therefore, quinoa bread aroma could be considered to be strong and pleasant both in terms of perceived aroma during baking (on-line experiment of volatiles released to the oven) and flavour during eating (static experiment of the aroma of the final bread).

Thus, it can be generally concluded that the higher release of volatile compounds from fermentation, lipids oxidation, esters, and furan derivatives from flour-based breads to the oven was related to their higher content in the corresponding breads. Only the release of pyrazine derivatives from starch-based breads was more related to the flowing out of volatile compounds during the moisture loss in the baking step. Interestingly, the losses of volatile compounds to the oven during baking have been generally higher in gluten-free breads than in wheat bread, which can undoubtedly be considered as one of the main findings of this study in order to justify the weaker aroma of gluten-free breads. Therefore, the baking conditions could be essential to control and keep a more intense flavour in gluten-free breads. By all means, the releases of the volatile compounds from cereal breads to the oven did not mean the depletion of aroma in the corresponding bread (based on data from the literature), although further sensorial experiments need to be done in order to corroborate this finding. Additionally, the leakage of volatile compounds during toasting followed the same pattern of those breads that presented the higher content at 40 min. of baking. Thus, ‘starch’ breads presented the higher release during toasting for fermentation, lipids oxidation, hexanoic acid, and Maillard compounds, quinoa bread presented the higher releases for acetic acid and acetoin and wheat bread for furan derivatives.

On this basis, PTR/MS clearly demonstrated its capability in the real-time monitoring of volatile compounds that were released to the oven during thermal processes, which can be beneficial for the analysis of a wide range of foods, including not only cereal products, but also other types of toasted and roasted foodstuffs.

## 4. Conclusions

The understanding of the generation and release of volatile compounds during baking could be crucial in explaining the residual volatiles remained in the final crumb/crust, which define the perceived flavour by the consumer. Different patterns were observed during the PTR-ToF-MS on-line monitoring of the baking vapours of five different gluten-free breads, including (1) fermentation, esters, and lipids oxidation compounds, which were demonstrated to be mainly generated before baking and presented a bell-shaped curve, (2) hexanoic acid and compounds generated both by fermentation and Maillard reactions, which followed an exponential plateau, and (3) Maillard and caramelisation compounds, which started to be exponentially released after 20 min (furan derivatives) or 30–35 min (pyrazine derivatives). In this process, fermentation compounds, acetic acid, and acetoin were released in higher ratios in ‘quinoa’ bread, lipids oxidation compounds in ‘rice’ bread, hexanoic acid in ‘teff’ bread, and furan derivatives in ‘wheat’ bread, which was in accordance with their higher content reported in the aroma of the final bread. Surprisingly, pyrazine derivatives and 2-acetyl-1-pyrroline were released in higher levels in ‘starch’ breads, which was attributed to the flowing out of volatile compounds due to moisture loss. Toasting promoted the substantial generation and/or release of volatile compounds from Maillard and caramelisation reactions. On this basis, it is demonstrated that the baking and toasting of bread clearly implied losses of volatile compounds from gluten-free breads, commonly higher than for wheat bread. Thus, studies that were related to the baking conditions (i.e., temperature, time, and moisture) could help to promote a more intense aroma in the final gluten-free baked product.

## Figures and Tables

**Table 1:** 54 volatile compounds selected as important contributors to bread aroma, including their *m*/*z* and formula. Thirty-nine volatile compounds were confirmed by means of fast GC-proton transfer reaction-time of flight-mass spectrometry (PTR-ToF-MS) using pure standards, while the other 15 were tentatively identified based on their *m*/*z*, possible formula, and literature about bread aroma.

Volatile Compound	*m*/*z*	Formula	Identification
3-Methyl-1-butanol	71.0856	C_5_H_11_^+^	Standard (1)
2-Methyl-1-butanol			Standard (2)
1-Pentanol			Standard (3)
Hexanal	83.0863	C_6_H_11_^+^	Standard (4)
1-Hexanol	85.1010	C_6_H_13_^+^	Standard (5)
Furfural	97.0288	C_5_H_5_O_2_^+^	Standard (6)
5-Methylfurfural	111.0451	C_6_H_7_O_2_^+^	Standard (7)
Pyrazine	81.0369	C_4_H_5_N_2_^+^	Standard (8)
2,3-Butanedione	87.0443	C_4_H_7_O_2_^+^	Standard (9)
Phenylacetaldehyde	121.0680	C_8_H_9_O^+^	Standard (10)
2-Ethyl-3-methylpyrazine	123.0826	C_7_H_11_N_2_^+^	Standard (11)
2-Acetylpyrazine			Standard (12)
2-Ethylpyrazine	109.0711	C_6_H_9_N_2_^+^	Standard (13)
2,3-Dimethylpyrazine			Standard (14)
2,5-Dimethylpyrazine			Standard (15)
2,6-Dimethylpyrazine			Standard (16)
Benzyl alcohol		C_7_H_9_O^+^	Standard (17)
Butanoic acid	89.0599	C_4_H_9_O_2_^+^	Standard (18)
Heptanal	97.1028	C_7_H_13_^+^	Standard (19)
1-Octen-3-ol	111.1181	C_8_H_15_^+^	Standard (20)
2-Acetyl-1-pyrroline	112.0759	C_6_H_10_NO^+^	Standard (21)
2,3-Diethylpyrazine	137.0983	C_8_H_13_N_2_^+^	Standard (22)
Nonanal	125.1339	C_9_H_17_^+^	Standard (23)
2,4-(E,E)-Decadienal	135.1221	C_10_H_15_^+^	Standard (24)
Ethyl octanoate	173.1577	C_10_H_21_O_2_^+^	Standard (25)
Ethyl hexanoate	145.1239	C_8_H_17_O_2_^+^	Standard (26)
Ethyl hexanoate	145.1239	C_8_H_17_O_2_^+^	Standard (27)
1-Methylpyrrol	82.0700	C_5_H_8_N^+^	Standard (28)
Limonene	137.1354	C_10_H_17_^+^	Standard (29)
3-Penten-2-ol	69.0702	C_5_H_9_^+^	Standard (30)
Benzaldehyde	107.0518	C_7_H_7_O^+^	Standard (31)
Hexyl acetate	145.1239	C_8_H_17_O_2_^+^	Standard (32)
Acetic acid	61.0280	C_2_H_5_O_2_^+^	Standard (33)
Furfuryl alcohol	81.0369	C_5_H_5_O^+^	Standard (34)
Acetoin	89.0599	C_4_H_9_O_2_^+^	Standard (35)
3-Methylbutanoic acid	103.0704	C_5_H_11_O_2_^+^	Standard (36)
2-Methylbutanoic acid	103.0704	C_5_H_11_O_2_^+^	Standard (37)
Phenylethyl alcohol	105.0721	C_8_H_9_^+^	Standard (38)
Hexanoic acid	117.0921	C_6_H_13_O_2_^+^	Standard (39)
Furan	69.0334	C_4_H_4_OH^+^	Tentative
3-Methylbutanal	69.0702	C_5_H_9_^+^	Tentative
2-Methylbutanal	69.0702	C_5_H_9_^+^	Tentative
2-Methylfuran	83.0498	C_5_H_7_O^+^	Tentative
5-Methyl-2(5H)-furanone	99.0446	C_5_H_7_O_2_^+^	Tentative
2-Methyl-6-propyl-pyrazine	137.0983	C_8_H_13_N_2_^+^	Tentative
Acetaldehyde	45.0325	C_2_H_5_O^+^	Tentative
Ethanol	48.0323	C_2_H_7_O^+^	Tentative
2,5-Diethylpyrazine	137.0983	C_8_H_13_N_2_^+^	Tentative
2,6-Diethylpyrazine			
3-Ethyl-2,5-dimethylpyrazine			
2-Ethyl-3,5-dimethylpyrazine			
5-Ethyl-2,3-dimethylpyrazine			
3-Ethyl-2,6-dimethylpyrazine			
2-Ethyl-3,6-dimethylpyrazine			

**Table 2:** Different patterns found among several gluten-free breads (quinoa, teff, rice and wheat flours and corn and wheat starches) and wheat control bread in the release of volatile compounds to the oven (headspace) measured by PTR-ToF-MS during 40 min. of baking at 200 °C.

Origin Based on the PTR-ToF-MS Pattern of Release of Volatile Compounds to the Oven during Baking	Possible Volatile Compounds
Fermentation origin	3-methyl-1-butanol, 2-methyl-1-butanol, 1-pentanol, 2,3-butanedione, 3-methylbutanal, 2-methylbutanal, acetaldehyde, ethanol
Lipids oxidation origin	1-hexanol, hexanal, heptanal, 1-octen-3-ol, nonanal, 2,4-decadienal, 3-penten-2-ol
Esters origin	ethyl octanoate, ethyl hexanoate and hexyl acetate
Acids origin	hexanoic acid
Fermentation-Maillard high retention in bread origin	phenylethyl alcohol, phenylacetaldehyde, benzaldehyde, benzyl alcohol, 3-methyl-butanoic acid and 2-methyl-butanoic acid
Fermentation-Maillard low retention in bread origin	acetic acid, butanoic acid and acetoin
Early Maillard origin	1-methyl-pyrrol, limonene, 2,3-diethyl-5-methylpyrazine, 2-ethyl-3-methylpyrazine, 2-acetylpyrazine, 2,3-diethylpyrazine, 2,5-diethylpyrazine, 2,6-diethylpyrazine, 3-ethyl-2,5-dimethylpyrazine, 2-ethyl-3,5-dimethylpyrazine, 5-ethyl-2,3-dimethylpyrazine, 3-ethyl-2,6-dimethylpyrazine and 2-ethyl-3,6-dimethylpyrazine
Late Maillard origin	2-acetyl-1-pyrroline, 2-ethylpyrazine, 2,3-dimethylpyrazine, 2,5-dimethylpyrazine and 2,6-dimethylpyrazine
Caramelization-Maillard origin	furan, furfuryl alcohol, furfural, 5-methyl-2(5H)-furanone, 5-methyl-furfural, 2-methylfuran

## Supplementary Materials

The following are available online at https://www.mdpi.com/2304-8158/9/10/1498/s1, Table S1: Fast GC-PTR-ToF-MS parameters of the 39 volatile compounds standards selected as important contributors to bread aroma, including the *m*/*z* and retention times.

## Abbreviations

E/N	electric field to gas density ratio
fast-GC	fast gas chromatography
FC	flow controller
HPMC	hydroxy propyl methyl cellulose
*m*/*z*	mass to charge ratio
PTFE	polytetrafluoroethylene
PTR-ToF-MS	proton transfer reaction-time of flight-mass spectrometry
sccm	standard cubic centimetres per minute
