# Supplementary material for: Real-Time Monitoring of Volatile Compounds Losses in the Oven during Baking and Toasting of Gluten-Free Bread Doughs: A PTR-MS Evidence
Source: Foods. 2020 Oct 20;9(10):1498. doi: 10.3390/foods9101498 (PMC7588997; doi:10.3390/foods9101498)
Supplement: Supplementary file 1 [file foods-09-01498-s001.pdf]

Real-time monitoring of volatile compounds losses in the oven during baking and toasting of gluten-free bread doughs: a PTR-MS evidence

Joana Pico [a,1*], Iuliia Khomenko [b], Vittorio Capozzi [c], Luciano Navarini [d], Franco Biasioli [b]

[a] I.U. Cinquima, Analytical Chemistry Group, University of Valladolid, Paseo de Belén Street 7, 47011 Valladolid, Spain.

[b] Research and Innovation Centre, Fondazione Edmund Mach, via E. Mach 1, San Michele all'Adige (TN), Italy

[c] Institute of Sciences of Food Production, National Research Council (CNR), c/o CS-DAT, Via Michele Protano, 71121 Foggia, Italy.

[d] Illycaffè S.p.a, Via Flavia, 110, 34147, Trieste (Italy).

*Corresponding author: Joana Pico

[1] Current affiliation: Wine Research Center, Applied Biology Department, The University of British Columbia, 2205 East Mall, V6T 1Z4, Vancouver, BC, Canada

e-mail: joana.picocarbajo@ubc.ca

Table Supplementary 1. Fast GC-PTR-ToF-MS parameters of the 39 volatile compounds standards selected as important contributors to bread aroma, including the m/z and retention times.

Volatile compound	m/z	Ret Time (s)
3-Methyl-1-butanol	71.0856	84.7
2-Methyl-1-butanol		83.7
1-Pentanol		93.0
Hexanal	83.0863	90.1
1-Hexanol	85.1010	100.3
Furfural	97.0288	107.0
5-Methylfurfural	111.0451	111.7
Pyrazine	81.0369	95.9
2,3-Butanedione	87.0443	80.3
Phenylacetaldehyde	121.0680	112.7
2-Ethyl-3-methylpyrazine	123.0826	100.1
2-Acetylpyrazine		114.6
2-Ethylpyrazine	109.0711	102.0
2,3-Dimethylpyrazine		103.6
2,5-Dimethylpyrazine		99.5
2,6-Dimethylpyrazine		102.3
Benzyl alcohol		99.5
Butanoic acid	89.0599	119.1
Heptanal	97.1028	89.3
1-Octen-3-ol	111.1181	96.7
2-Acetyl-1-pyrroline	112.0759	105.0
2,3-Diethylpyrazine	137.0983	101.9
Nonanal	125.1339	104.3
2,4-(E,E)-Decadienal	135.1221	119.1
Ethyl octanoate	173.1577	100.5
Ethyl hexanoate	145.1239	93.0

Table Supplementary 1. Continued

Volatile compound	m/z	Ret Time(s)
Ethyl hexanoate	145.1239	93.0
1-Methylpyrrol	82.0700	85.6
Limonene	137.1354	89.3
3-Penten-2-ol	69.0702	96.7
Benzaldehyde	107.0518	104.2
Hexyl acetate	145.1239	104.2
Acetic acid	61.0280	nd*
Furfuryl alcohol	81.0369	nd*
Acetoin	89.0599	nd*
3-Methylbutanoic acid	103.0704	nd*
2-Methylbutanoic acid	103.0704	nd*
Phenylethyl alcohol	105.0721	nd*
Hexanoic acid	117.0921	nd*

* nd means "not detected"

© 2020 by the authors. Licensee MDPI, Basel, Switzerland. This article is an open access article distributed under the terms and conditions of the Creative Commons Attribution (CC BY) license (http://creativecommons.org/licenses/by/4.0/).
